# Analysis of the efficacy of a single subumbilical stoma for bilateral cutaneous ureterostomy after radical cystectomy

**DOI:** 10.1186/s40001-023-01250-z

**Published:** 2023-08-07

**Authors:** Zhenyu Fu, Zhen Tian, Yongchang Chen, Zongming Jia, Chengyu Wang, Xuefeng Zhang, Weijie Zhang, Gang Li, Xuedong Wei, Yuhua Huang

**Affiliations:** 1 Department of Urology, the First Affiliated Hospital of Soochow University, Suzhou, Jiangsu China; 2 Department of Urology, Changshu No. 2 People’s Hospital, Changshu, Jiangsu China

**Keywords:** Bladder cancer, Radical cystectomy, Ureterostomy with a single stoma, Efficacy, Complications

## Abstract

**Background:**

Radical cystectomy and urinary diversion are the standard surgical treatments for patients with muscle-invasive or high-risk, or recurrent non-muscle-invasive bladder cancer. Although this approach significantly prolongs patient survival, it can lead to postoperative complications. This study aims to compare the efficacy and complications of bilateral cutaneous ureterostomy with a single subumbilical stoma to those of cutaneous ureterostomy with two stomas and an ileal conduit as a means of urinary diversion after radical cystectomy. The findings of this study will provide valuable information for healthcare providers in selecting the appropriate urinary diversion method for their patients.

**Methods:**

The clinical data for 108 patients who received bilateral cutaneous ureterostomy with a single subumbilical stoma (ureterostomy with a single stoma group), cutaneous ureterostomy with two stomas (ureterostomy with two stomas group), or an ileal conduit (ileal conduit group) after radical cystectomy were retrospectively analysed. The operative time, pathological stage, survival status, perioperative complication rate, rate of successful first extubation, rehospitalization rate at 6 months after surgery,ostomy-related medical costs,and postoperative quality of life were compared between the three groups of patients.

**Results:**

A significant difference in the operative time was found between the three groups (*P* = 0.001). No significant differences in pathological stage, survival status, perioperative complication rate, rehospitalization rate at 6 months after surgery, or bladder cancer index (BCI) score were identified among the three groups. The difference in the successful first extubation rate between the three groups of patients was significant (*P* = 0.001). Significant differences in ostomy-related medical costs were observed among the three groups of patients (*P* = 0.006).

**Conclusion:**

A single subumbilical stoma for bilateral cutaneous ureterostomy after radical cystectomy may result in shorter surgery time, increased success rates for initial catheter removal, and lower medical expenses. However, to confirm these findings, further prospective randomized clinical trials are necessary.

## Introduction

Globally, bladder cancer is among the top 10 most common malignancies, and causes enormous social burden [1]. Various treatments for bladder cancer are available [2, 3], but for muscular invasive bladder cancer, radical cystectomy plus urinary diversion is one of the most important methods [4, 5]. A common dilemma in high-risk patients undergoing radical cystectomy is the decision of what kind of urinary diversion to perform [6]. There are several methods available,such as ileal conduit and cutaneous ureterostomy, among others, but the ultimate goal of treatment is to protect renal function and improve the patient's quality of life [7].

The ileal conduit technique, also known as the Bricker procedure, has been used for over 50 years and is considered a safe and straightforward method for urinary diversion. It is frequently utilized after radical cystectomy, a surgical procedure where the bladder is removed, to treat bladder cancer [8]. In clinical practice, urinary diversion using the ileal conduit is a common procedure performed after radical cystectomy, primarily due to the easy accessibility of the donor intestine and the convenience of a single stoma for nursing care. However, this surgical procedure can have a negative impact on the integrity of the small intestine and can be more complicated compared to other options [6]. Additionally, there is a higher risk of postoperative intestinal complications, and long-term complications such as morphological changes in the upper urinary tract, parastomal hernia, and metabolic acidosis may occur [9]. Furthermore, if anastomotic stenosis of the ureteral and ileal conduit occurs, treatment can become more challenging [10, 11].

Another common method of altering the flow of urine is through a surgical procedure known as cutaneous ureterostomy. This surgical procedure is available in two types: the two-stoma type and the single-stoma type. Compared to other surgical procedures, cutaneous ureterostomy offers several benefits such as a simple operation, shorter operative time, reduced intraoperative blood loss, and a shortened hospital stay [12]. However, the traditional method of cutaneous ureterostomy is susceptible to complications such as stenosis and retrograde infection of the upper urinary tract [13, 14]. These complications act as barriers to the widespread adoption of this surgical approach.

We have reason to believe that the technology used in surgery can be optimized, so we have developed an improved technique that can better utilize the advantages of ileal conduit and ureterocutaneostomy, thereby reducing the difficulty of the surgical procedure and minimizing damage to the patient. The objective of this study is to compare the incidence of early and late complications, treatment effectiveness, medical expenses, and quality of life among three groups of bladder cancer patients: the single stoma group, the two stomas group, and the ileal conduit group.

## Patients and methods

### General information

**Fig. 1:**
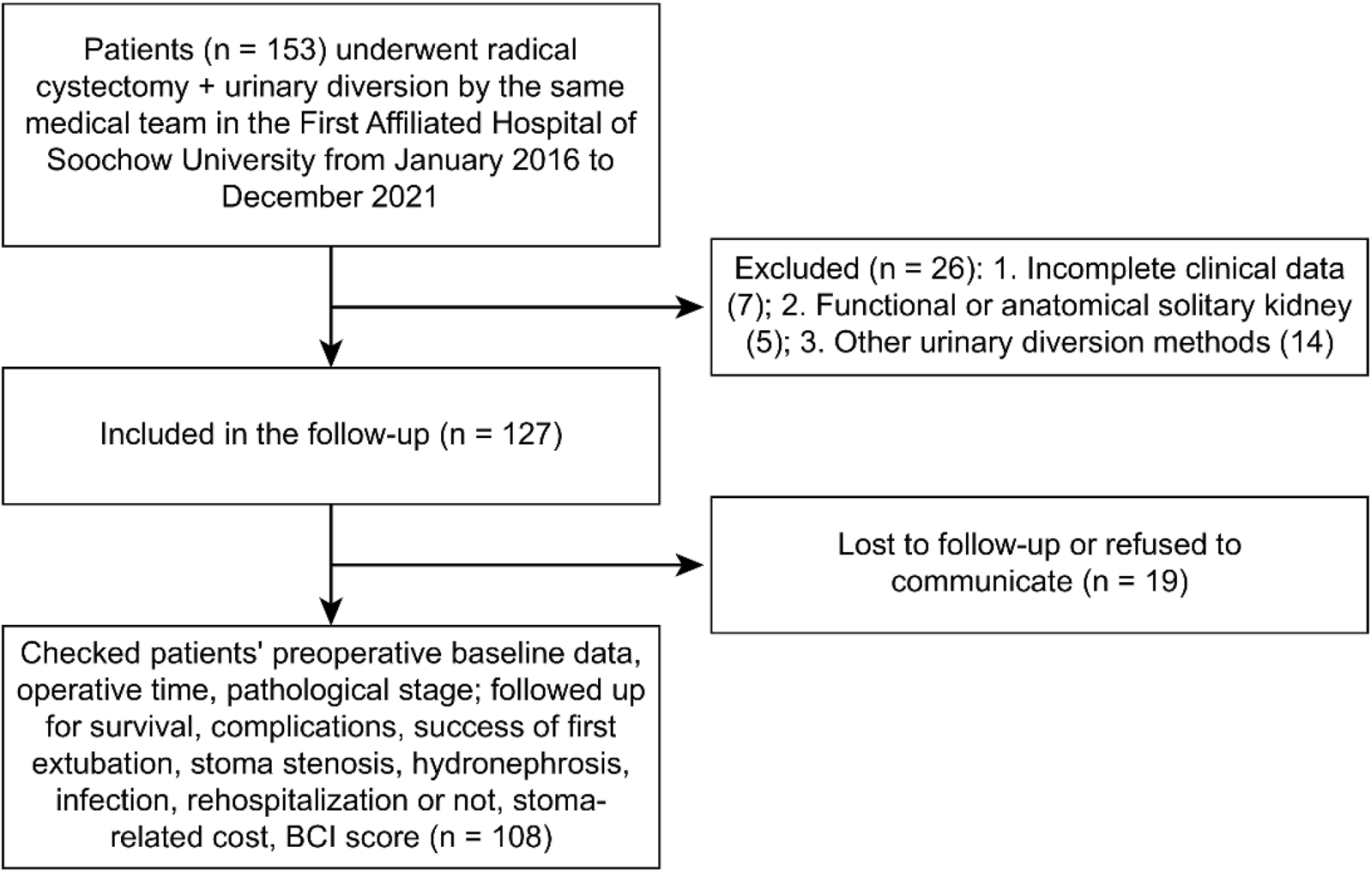
Flowchart of patient selection

A total of 153 patients were included in the study: 26 were excluded at first, 127 entered the follow-up, and 19 were lost to follow-up, leaving 108 patients for analysis (Fig. 1). This study retrospectively analysed the clinical data for patients who underwent bilateral cutaneous ureterostomy with a single subumbilical stoma after radical cystectomy (ureterostomy with a single stoma group) from January 2016 to December 2021. The data for patients undergoing cutaneous ureterostomy with two stomas (ureterostomy with two stomas group) or with an ileal conduit (ileal conduit group) after radical cystectomy over the same period were collected to compare the efficacy, complications, and quality of life.

### Ureterostomy with a single stoma

**Fig. 2:**
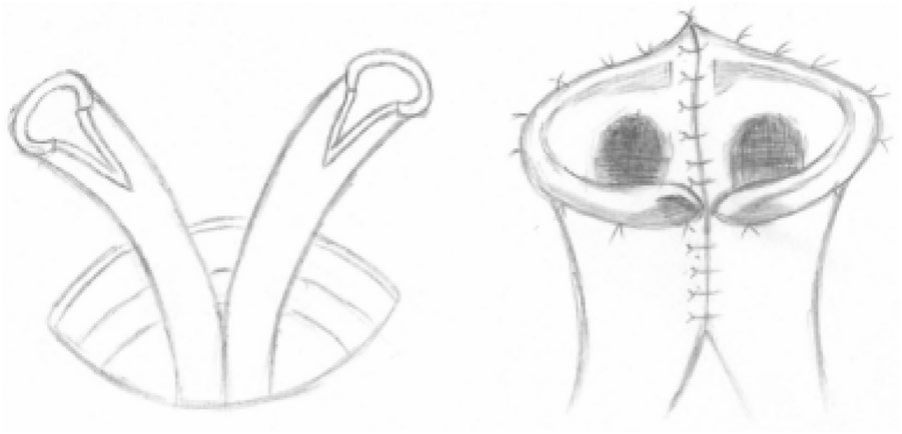
(Left) This schematic diagram depicts the ureters being pulled out of the abdominal wall and a lateral incision being made on the ureter. (Right) This schematic diagram shows the effect of a ureteral anastomosis. As described in the reference provided, the procedures for performing ureterostomy with two stomas and ileal conduit surgery were followed. [15, 16]

Single stoma: A circular incision with a diameter of approximately 1.5 cm was made 5 cm below the umbilicus. A longitudinal incision was made on both sides of the ureter adjacent to the wall of the ureter, approximately 8–10 mm, in a V-shaped incision. Lateral anastomosis with a V-shaped incision was performed at the incision sides, and the V-shaped incision was merged to form a nipple shape. One stent tube was placed in each of the two ureters through the nipple. Intermittent sutures secured the nipple to the skin (Fig. 2).

### Postoperative follow-up

The ureteral stent was removed 6 months after the surgery in the ureterostomy with a single stoma group and the ureterostomy with two stomas group and 3 months after the surgery for the ileal conduit group.The patients who were rehospitalized due to surgery-related complications (including ureteral stenosis, hydronephrosis, and pyelonephritis) 6 months after surgery were followed up. The medical expenses related to the stoma (including ostomy bag, ureteral stent, and nursing) were recorded. The Bladder Cancer Index (BCI) [17], which is used to assess the quality of life of bladder cancer patients after surgery, was completed either 1 year after the surgery or, if the follow-up period was shorter than 1 year, at the last follow-up.The severity of complications was graded according to the Clavien‒Dindo grading system [18].

### Statistical analysis

SPSS 27.0 (IBM Corp., Armonk, NY, USA) was used to process the data. The normality of the data was assessed using the Shapiro–Wilk test (SW). Normally distributed numerical variables were expressed as mean ± standard (x̄ ± s), while skewed numerical variables were expressed as median (interquartile range), abbreviated as M (P25, P75). Categorical data are presented as numbers (%). Differences were compared by analysis of variance and the rank sum test for measurement data and the chi-squared test for count data, except that when T < 5, Fisher’s exact probability method was used. *P* < 0.05 was considered statistically significant.

### Ethics statement

The procedure was in line with the Declaration of Helsinki of the World Medical Association revised in 2013. This study was approved by the Medical Ethics Committee of the First Affiliated Hospital of Soochow University (Ethics number: 2022–363). All patients signed informed consent for surgery.

## Results

After analyzing the preoperative baseline data of the patients, it was found that there were significant differences in age and the American Society of Anaesthesiologists (ASA) score among the three groups (*P* = 0.001). ASA score is a system that is commonly used to assess the physical status of a patient before undergoing anesthesia or surgery [12]. However, there were no significant differences noted in sex, body mass index (BMI), hypertension, or diabetes among the three groups (*P* > 0.05, Table 1).

**Table 1:** Comparison of the patients’ preoperative clinical data

Variable	Single stoma group (27)	Two stomas group (45)	Ileal conduit group (36)	*P* value
Sex				0.703
Male	24 (85.2)	41 (91.1)	31 (86.1)	
Female	3 (14.8)	4 (8.9)	5 (13.9)	
Age [years]	71.9 ± 9.4	70.1 ± 10.1	63.6 ± 7.3	0.001
BMI [kg/m^2^]	22.9 ± 3.2	23.3 ± 3.8	24.1 ± 3.2	0.392
Hypertension				0.326
Yes	14 (51.9)	25 (55.6)	14 (38.9)	
None	13 (48.1)	20 (44.4)	22 (61.1)	
Diabetes				0.246
Yes	7 (25.9)	5 (11.1)	5 (13.9)	
None	20 (74.1)	40 (88.9)	31 (86.1)	
ASA score				0.001
1	15 (55.6)	24 (53.3)	34 (94.4)	
2	11 (40.7)	20 (44.4)	2 (5.6)	
3	1 (3.7)	1 (2.2)	0 (0)	

n (%): number of cases (percentage); x̄ ± s: mean ± standard deviation

All 108 patients successfully completed the surgery (operative times and postoperative data are shown in Table 2.

**Table 2:** Comparison of the patients’ surgical and postoperative clinical data

Variable	Single stoma group (27)	Two stomas group (45)	Ileal conduit group (36)	*P* value
Operative time [min]	240 (180, 354)	246.5 (219.3, 303.8)	350 (302.5, 446.3)	0.001
Pathological T stage				
T1	1 (3.7)	6 (13.3)	5 (13.9)	
T2	20 (74.1)	29 (64.4)	26 (72.2)	
T3	1 (3.7)	4 (8.9)	1 (2.8)	0.692
T4	5 (18.5)	6 (13.3)	4 (11.1)	
Pathological N stage				
N1	7 (25.9)	10 (22.2)	7 (19.4)	0.803
N0	20 (74.1)	35 (77.8)	29 (80.6)	
Complications	11 (40.7)	13 (28.9)	14 (38.9)	
Yes	16 (59.3)	32 (71.1)	22 (61.1)	0.506
None				
Successful first extubation				
Yes	20 (74.1)	2 (4.4)	36 (100)	
No	7 (25.9)	43 (95.6)	0 (0)	0.001
Hospitalization after 6 months of discharge				
Yes	5 (18.5)	10 (22.2)	8 (22.2)	
None	22 (81.5)	35 (77.8)	28 (77.8)	0.92
Ostomy-related costs	298.5 (224.2, 350.6)	405.4 (307, 604.4)	305 (218.6, 404.7)	0.006
BCI score				
Urinary function	88.3 (88.3, 88.3)	85 (68.4, 88.3)	85 (85, 88.3)	0.399
Urinary troubles	80 (73.1, 90.6)	73.1 (73.1, 90.6)	73.1 (73.1, 88)	0.516
Intestinal function	85 (78.8, 93.8)	85 (78.8, 93.8)	85 (62.5, 88.8)	0.261
Intestinal troubles	95.8 (91.7, 95.8)	91.7 (66.9, 95.8)	91.7 (87.5, 95.8)	0.05

M (P25, P75): median (25%, 75%); n (%): number of cases (percentage)

**Fig. 3:**
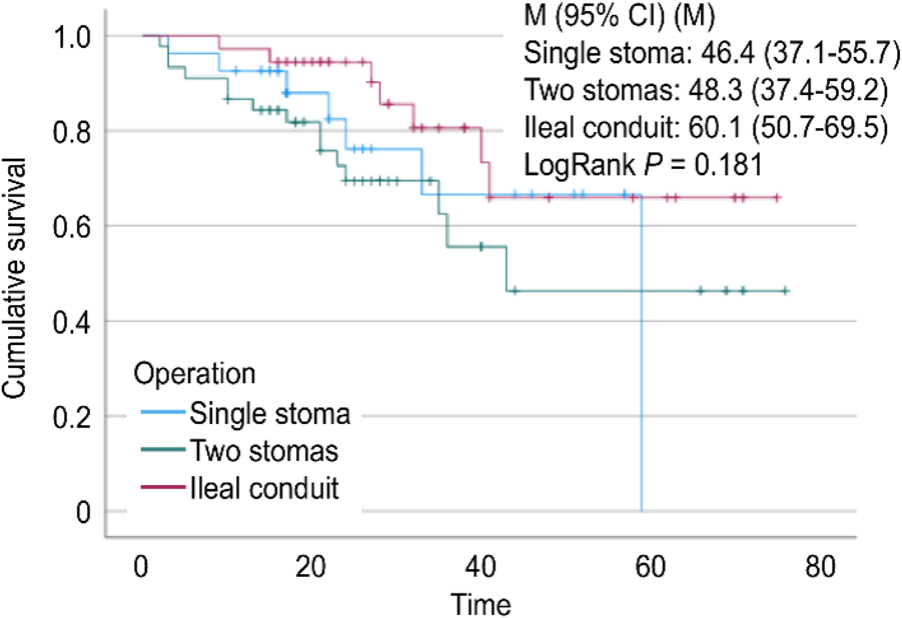
Survival curve of patients

No perioperative deaths occurred. The median follow-up time was 22 (16, 44) months in the single-stoma group. Seven deaths occurred, including six deaths due to tumour progression and one death due to a traffic accident. In the two-stoma group, the median follow-up time was 24 (16, 32) months, and 15 deaths occurred, including 12 deaths due to tumour progression and three deaths due to cardiovascular and cerebrovascular diseases. The median follow-up time was 29 (21, 41) months in the ileal conduit group, with seven patients dying from tumour progression. No difference in the mortality rate was found among the three groups (*P* > 0.05, Fig. 3).

The rate of successful first extubation in the single-stoma group was 74.1% (20/27). The success rate of the first extubation in the single-stoma group was significantly higher than that in the two-stoma group (*P* = 0.001).

A significant difference in the average monthly ostomy-related medical expenses was found between the three groups (*P* = 0.006), and the medical expenses of the single-stoma group were significantly lower than those of the two-stoma group (*P* = 0.006).

No significant difference in urinary function, urinary trouble, intestinal function, or intestinal trouble was noted between the three groups (*P* > 0.05). All patients answered the questionnaire saying they had an inactive sex life and reported no sexual worries.

## Discussion

The objective of this study was to find a urinary diversion method that is simple and effective with minimal complications. To achieve this goal, an improved urinary diversion technique was proposed, which could increase the catheter-free rate and potentially reduce the occurrence of stomal stenosis. The study showed that the ureterostomy with a single stoma group had a shorter surgical time compared to the ileal conduit group and no patient died during the perioperative period. Additionally, the ureterostomy with a single stoma group had a higher success rate for the first extubation and lower ostomy-related medical expenses compared to the ureterostomy with two stomas group. Furthermore, the mortality rate was similar between patients who underwent this technique and those who underwent the other two urinary diversion methods during the follow-up period.

1. The occurrence of perioperative complications. The most common complications after radical cystectomy are gastrointestinal complications, infection, trauma-related problems, and urogenital defects [19–21]. Additionally, it is important to note that perioperative complications not only lead to prolonged hospital stay, increased medical costs, and decreased quality of life for patients, but also pose a significant burden on healthcare resources. Therefore, it is crucial to carefully evaluate the benefits and risks of different surgical methods and to identify strategies to minimize perioperative complications in patients undergoing radical cystectomy. There is literature to suggest that the choice of surgical method is closely linked to the incidence of complications. In particular, the ileal conduit method has been associated with the highest rates of severe complications, as well as surgical reinterventions and late complications affecting the intestinal tract [22].Our observation showed that the overall incidence of intestinal obstruction and infection was similar in the three groups. Since patients undergoing ureterostomy are older and have an increased risk of anaesthesia, ureterostomy may be safer than ileal conduit during the perioperative period.
2. The occurrence of long-term postoperative complications. Ureteral anastomotic stricture is a frequently encountered and particularly difficult postoperative complication in patients who have undergone diverted urinary flow surgery. The incidence of ureteral anastomotic stenosis is 8% to 22%, and ureteral and intestinal anastomotic stenosis can lead to hydroureter and renal dysfunction, as well as recurrent urinary tract infections [23, 24]. Long-term placement of a stent tube is often required to prevent ureteral stenosis after cutaneous ureterostomy but is prone to retrograde infection, which increases medical costs [25]. Our observations indicated that the rate of successful first extubation after surgery in the two-stoma group was 4.4% (2/45), and the vast majority of patients required long-term indwelling ureteral stents. The success rate of successful first extubation in the single-stoma group was 74.1% (20/27). After extubation, most patients did not have ureteral stenosis or retrograde infection and no longer needed an indwelling ureteral stent. Compared to other techniques, the creation of a single stoma during surgery can significantly increase the chances of successfully removing a ureteral stent while not raising the risk of ureteral anastomotic stricture. This advantage highlights the significance of this procedure.
3. Analysis of ostomy-related costs. Patients with bladder cancer have relatively high medical costs after surgery [26], including ostomy-related costs, chemotherapy, and basic disease treatment costs. We compared the average monthly medical expenses related to the ostomy between the three groups of patients, and the medical expenses of the single-stoma group were significantly lower than those of the two-stoma group. Compared with that in the two-stoma group, the proportion of indwelling ureteral stents in the single-stoma group was lower, which reduced the likelihood of retrograde infection in the patients and reduced their economic burden.
4. Postoperative quality of life. The type of urinary diversion does not affect overall survival, and the long-term quality of life is similar [16, 27]. However, unilateral ureterostomy has also been reported to require fewer ostomy bags, impose lower nursing, financial, and psychological burdens on the patient, and improve their mental state [28]. A meta-analysis [29] using the BCI to assess the quality of life of patients after radical cystectomy indicated that the long-term results of urinary function were significantly better and urinary distress was significantly less in the ileal conduit group than in the orthotopic neobladder group despite no differences in bowel function or distress. Sexual function could not be accurately assessed. BCI evaluation also revealed no significant differences in urinary function, urinary difficulty, intestinal function, or intestinal trouble among the three groups of patients. All patients reported no sex life or sexual troubles after surgery; therefore, the sexual function domain was invalid. This phenomenon may be related to the fact that most elderly individuals in China are more conservative in their sexuality, have a lower sexual demand, and experience greater postoperative psychological pressure.

## Limitations

The sample sizes of our three groups were small and might not adequately reflect the overall population. Due to the small sample size, the propensity score method could not be used to reduce the selection biases in age and ASA score. This was a single-centre study; therefore, the patient population was not representative. We hope to further observe the efficacy and complications of patients through prospective, multicentre, controlled studies in the future.

## Conclusion

For patients undergoing urinary diversion after radical cystectomy, the survival rate and the incidence of complications were similar in patients receiving bilateral cutaneous ureterostomy with a single subumbilical stoma, cutaneous ureterostomy with two stomas, or an ileal conduit. No difference was found in the percentage of rehospitalization at 6 months after surgery or in the BCI scores. However, the intestinal disturbance rate in the single-stoma group was lower than that in the ileal conduit group, and the operative time was shorter; compared with those of patients with two stomas, medical expenses related to ostomy care were lower in the single-stoma group, and the percentage of successful first extubation was significantly increased. Radical cystectomy with bilateral cutaneous ureterostomy with a single subumbilical stoma is a recommended alternative for urinary diversion to the ileal conduit and cutaneous ureterostomy with two stomas.

## Data Availability

FZY had full access to all the data in the study and assumes responsibility for the integrity of the data and the accuracy of the data analysis.
